# Prognostic Relevance of PDL1 and CA19-9 Expression in Gallbladder Cancer vs. Inflammatory Lesions

**DOI:** 10.3390/curroncol30020121

**Published:** 2023-01-25

**Authors:** Neetu Rawal, Supriya Awasthi, Nihar Ranjan Dash, Sunil Kumar, Prasenjit Das, Amar Ranjan, Anita Chopra, Maroof Ahmad Khan, Sundeep Saluja, Showket Hussain, Pranay Tanwar

**Affiliations:** 1 Laboratory Oncology Unit, Dr. B.R.A. Institute-Rotary Cancer Hospital, All India Institute of Medical Sciences, New Delhi 110029, India; 2 Department of GI Surgery, All India Institute of Medical Sciences, New Delhi 110029, India; 3 Department of Surgical Oncology, Dr. B.R.A. Institute-Rotary Cancer Hospital, All India Institute of Medical Sciences, New Delhi 110029, India; 4 Department of Pathology, All India Institute of Medical Sciences, New Delhi 110029, India; 5 Department of Biostatistics, All India Institute of Medical Sciences, New Delhi 110029, India; 6 Department of GI Surgery, GB Pant Hospital, New Delhi 110002, India; 7 Divison of Molecular Oncology, National Institute of Cancer Prevention & Research, NICPR-ICMR, Noida 201301, India

**Keywords:** programmed cell death-1 ligands (PDL1), gallbladder cancer (GBC), enzyme-linked immunosorbent assay (ELISA), real time-PCR, inflammatory lesion, immunotherapy

## Abstract

Chronic inflammation in the gallbladder leading to persistent epithelium damage promotes invasive cancer. The study aimed to assess the prognostic value of PDL1 and CA19-9 markers in cancer/inflammatory lesions of the gallbladder. A total of 29 cases (19 cancer and 10 inflammatory) were included. The PDL1 protein concentration level and mRNA expression were assessed in the tissues’ lysates by ELISA and real-time PCR, respectively. PDL1 and CA19-9 concentration levels were compared and statistically related with clinico-pathological variables. The PDL1 protein level and its relative mRNA expression were correlated. Kaplan–Meir survival and Cox regression analyses were conducted for predicting prognosis. This study investigated the PDL1 and CA19-9 marker expression in both cancer and inflammatory cases of the gallbladder (*p* = 0.48 and *p* = 0.17 respectively). PDL1 protein expression was significantly associated with the hormonal profile of the cases (*p* = 0.04) at an optimum cut-off value of 13 pg/mL, while the CA19-9 marker expression was correlated with the status of liver metastasis (*p* = 0.0043) and size of the tumor (*p* = 0.004). A low PDL1 concentration was found when compared to the CA19-9 level among cancer cases (*p* = 0.12) and proportional in the inflammatory lesions (*p* = 0.63). A significant positive correlation was found between the PDL1 protein and its relative mRNA expressions in the inflammatory lesions (*p* = 0.029) when compared to cancer cases (*p* = 0.069). Our results showed that a protein-based assay for PDL1 expression would be more sensitive compared to RNA based assays for GBC risk stratifications. Overall survival was predicted with CA19-9 and PDL1 levels (*p* = 0.0074, *p* = 0.23, respectively). PDL1 and CA19-9 may act as a probable predictor of a poor prognosis in gallbladder cancer (GBC) cases.

## 1. Introduction

Gallbladder carcinoma (GBC) is a relatively uncommon but aggressive malignancy and has a 5-year survival of less than 5%. Survival may be reached upto 75% if proper treatment is performed [1,2]. The most important risk factor in developing gallbladder cancer is gallstones, which cause chronic inflammation in the form of cholecystitis [2]. About 1% of GBC is detected incidentally during elective cholecystectomy for cholelithiasis. Complete radical extirpation is the only effective surgical modality for GBC, especially if it is limited to mucosa or submucosa. However, unfortunately, about 90% of GBC presents to clinics at a very advanced stage [3]. About 80 to 97% of GBCs are adenocarcinoma. Other gallbladder cancer subtypes are papillary, mucinous, squamous, and adenosquamous [4]. Papillary adenocarcinomas have a relatively better prognosis when compared to non papillary carcinomas [5]. The risk factors for gallbladder cancer are age, sex, ethnicity, gallstones, chronic inflammation, infections, heavy metal environmental exposures, obesity, gallbladder polyps, genetics, and pancreaticobiliary ductal junctional abnormality [6]. The association of gallstones with GBC has been observed since the 19th century. Various studies all over the globe have focused on detailed information on the persistent association of stone and GBC. Our country, northern India, has a 20-times higher incidence of symptomatic gallstones when compared to the southern region [7]. The cholesterol/mixed stone is usually found in the northern part compared with a predominance of pigmented stone in the southern part of India [7]. The formation of gallstones initiates a vicious cycle of a persistent trauma-induced inflammation stage followed by partial regeneration recurrent cycles in epithelial mucosa [8,9,10]. Patients with gallstone inflammatory lesions have a21- to 57-fold higher risk of the formation of GBC [11]. The GBC incidence in females is 2 to 6 times higher than in males [12]. The co-expression of estrogen and progesterone receptor in GBC is increased in females as compared to males and could be the potential treatment target [13]. CEA, CA125, CA242, and CA19-9 are the tumor markers used for the diagnosis of liver, gastric, colorectal, and pancreatic cancers. The abovementioned markers show relatively low sensitivity and specificity when tested for the diagnosis and follow-up for GBC. The diagnostic sensitivity and GBC prognosis improve with the battery of combined tumor markers [11,12]. Though tumor markers such as CA19-9 and CEA are extensively used in diagnosing GBC, the evidence of these two markers in predicting GBC metastasis is still lacking. As per the study by Wang et al. [13], when compared with the CEA marker, the CA19-9 value increases gradually with the progression of the clinical stage [13]. The tumor burden increases in the patients with the increase in CA19-9 value [14]. As per the Kang et al.study [14], CA19-9 and CEA markers are unsuitable for the detection of GBC from the normal control group of patients as they have high specificity and low sensitivity for early GBC [14]. Still, the diagnosis of GBC depends on a clinical examination followed by noninvasive imaging (fine needle aspiration cytology (FNAC)). There is no consistent tumor marker in clinical practice for the diagnosis and prognosis of GBC [15]. The overall prognosis of GBC is poor, and there is an impending need for additional therapeutic intervention in addition to radical surgeries. The targeted therapy on molecular receptors which are associated with tumor invasion, proliferation, and immune response in multiple cancers shows improved survival [16]. There is a significant increase in the demand for an immunomodulatory approach which targets the programmed death-ligand 1 (PDL1) and programmed cell death protein 1 (PDL1) interaction [17].The current study aimed to assess the prognostic value of the PDL1(targeted therapy) and CA19-9 markers in cancer/inflammatory lesions of the gallbladder with a possible primary objective of using such molecular receptor inhibitors as an adjunct treatment option.

## 2. Materials and Methods

### 2.1. Ethics, Consent, and Sample Collection

This study was approved by the institute ethics committee at the All India Institute of Medical Sciences, New Delhi, India (IECPG-608/25 November 2020), and protocols as per the principles of the Declaration of Helsinki. Informed consent was obtained at the time of screening from each patient at the GI surgery department, AIIMS, New Delhi. The history of the patient and clinical information were collected from the enrolled participants. The gallbladder tissue was collected at the time of surgery in a 1 × PBS vial and was stored at −80 °C. The diagnostic confirmation of the tumor and the inflammatory lesion was completed by routine histopathology. The clinico-pathological information and CA19-9 serum level were retracted from the clinical case records.

### 2.2. ELISA Assay

The protein lysate was prepared by the easy prep lysis buffer (Thermo Scientific, Waltham, MA, USA) from 29 gallbladder tissues (19 cancerous and 10 inflammatory lesions) and was assayed following the manufacturer’s protocol by a commercially available PDL1- ELISA Kit (Invitrogen, Thermo Catalog no. BM2212). Briefly, 96-well plates were incubated at room temperature with standard, sample, and blank in duplicate. After incubation, microwell plates were washed three times with the wash buffer. Then, 100 µL of diluted Biotin-Conjugate antibodies were added to all wells and incubated at room temperature (18 to 25 °C) for 1 h. After the washing step, 100 µL of diluted Streptavidin-HRP was added and incubated for 30 min. The color development took place by adding 100 µL of the TMB substrate solution to all the wells, and incubation at room temperature for 30 min. Then, 100 µL of the stop solution was added to all wells when the highest standard had developed a blue color. The absorbance of both the standards and samples were measured at 450 nm.

### 2.3. RT-PCR

Total RNA was extracted from 24 gallbladder tissues (17 cancer cases and 7 inflammatory controls) using the RNA later method (Qiagen RNAeasy mini kit) as per the manual’s instructions. cDNA was made by reverse transcription (Improm II RT system, Promega, Madison, WI, USA) followed by a quantitative PCR run using SYBR green dye and Oligo(dT) primer on the Agilent Mx3000P qPCR Platform (Agilent, Santa Clara, CA, USA). The specific primer sequences of PDL1 were Fwd: TGGCATTTGCTGAACGCATTT, Rev: AGTGCAGCCAGGTCTAATTGT, and *GAPDH* served as the housekeeping gene with the sequence of Fwd: AGGTCGGAGTCAACGGATTT, Rev: ATGAAGGGGTCATTGATGGCA. The 2^−∆Ct^ equation was used to calculate the relative mRNA expression of PDL1.

### 2.4. Statistical Analysis

The continuous variables were summarized using *x* ± *s* and the median (range). Student’s t-test was used to determine the PDL1/CA19-9 concentration in cancer cases and inflammatory lesions. The ROC curve analysis was used to calculate the optimal cut-off value for PDL1 concentration. The chi square test was used for the clinico-pathological variables’ correlation with PDL1 and CA19-9 levels, along with an association of gallstones among cancer and inflammatory cases. The PDL1 protein level and its corresponding RNA expression were correlated in cancer and inflammatory cases by a paired t-test. The Kaplan–Meier method with a log-rank test was used to predict overall survival. The univariate and multivariate Cox proportional hazard regression analysis was used to identify the association of prognostic factors with survival outcomes. All the statistical analyses were performed with the graphpad prism. *p* < 0.05 was considered statistically significant.

## 3. Results

### 3.1. PDL1 and CA19-9 Concentration in Cancer Casesand Inflammatory Lesion of Gallbladder

**Figure 1:**
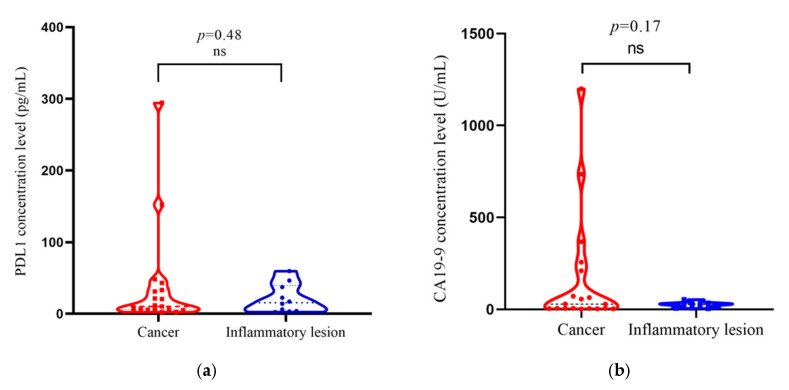
(**a**) Box violin plot shows PDL1 concentration (pg/mL) level in cancer cases (*n* = 19) and inflammatory lesions (*n* = 10), (*p* = 0.48); The red color dot represents PDL1 concentration level in individual cancer cases and blue color dot represents PDL1 concentration in individual inflammatory cases of gallbladder. (**b**) Box violin plot shows CA19-9 concentration (U/mL) level in cancer cases (*n* = 19) and inflammatory lesions (*n* = 10), (*p* = 0.13). Red color dot represents CA19-9 concentration level in individual cancer cases and blue color dot represents CA19-9 concentration level in individual inflammatory cases of gallbladder.

The overall data from the 29 patients (19 cancer cases and 10 inflammatory lesions) at AIIMS, New Delhi were analyzed. The PDL1 level in the tissue lysate was tested by ELISA. The mean value of the PDL1 level in gallbladder cancer cases was 37.67 ± 16.24 pg/mL, and in the inflammatory lesions was 21.43 ± 6.360 pg/mL. The PDL1 protein concentration was compared in two phenotypes of the gallbladder tissue (cancer and inflammatory lesion) (*p* = 0.48 as shown in Figure 1a). The presence of gallstones was significantly correlated among cancer cases and inflammatory lesions (*p* < 0.0001).The receiver operating characteristic (ROC) curve was plotted to determine the optimum PDL1 concentration for predicting PDL1 expression level (high/low).The area under the curve obtained was 0.5053 [95% confidence interval (95%CI): 0.2773 to 0.7333, *p* = 0.9634]. The optimum PDL1 concentration cut-off value of 13.03 pg/mL was determined by the Youden index formula [18] through the ROC curve and used for clinico-pathological correlation.

The CA19-9 concentration levels in serum present in the same set of patients (which included 19 cancer cases and 10 inflammatory lesions) were collected from clinical case records. The mean value of CA19-9 in cancer cases was 161.1 ± 71.48 U/mL, and in the inflammatory lesions was 23.93 ± 4.856 U/mL. CA19-9 concentration was compared in cancer cases and inflammatory lesions (*p* = 0.17 as depicted in Figure 1b). In our study, we used a validated optimum CA19-9 cut-off value of 37 U/mL (used in routine examination) [19] for the clinico-pathological correlation with CA19-9 concentration.

### 3.2. Clinico-Pathological Correlations of Gallbladder Cancer/Inflammatory Cases with PDL1 and CA19-9 Concentration Level

#### 3.2.1. PDL1

The clinico-pathological characteristics were tabulated in Table 1. The gallbladder cancer/inflammatory cohort included 13 males and 16 females with a median age of 52 years (range, 23–77 years). There were 11 (57.8%) cases diagnosed with tumor size (T1/T2), and 8 (42.1%) cases with (T3/T4). About 6 (31.5%) cases were presented with lymph node metastasis, and 13 (68.4%) were without any metastasis but had no significant (*p* = 0.59) association with PDL1 expression at the optimum cut-off level of 13 pg/mL. Similarly, 17/19 cases (89.4%) were morphologically characterized as well-differentiated adenocarcinoma and 2 (10.5%) were poorly differentiated/undifferentiated carcinoma in association withPDL1 expression (*p* = 0.079) at an optimum cut-off level. Additionally, about 6 (31.5%) cases were presented with local liver metastasis, and 13 (68.4%) had no evidence of metastasis. The PDL1 concentration level among both cancerous as well as inflammatory lesions was found to be above the optimum cut-off value in 14/29 cases (48.2%), whereas the PDL1 concentration level found was below the optimum cut-off value in the remaining 15 cases (51.7%). A significant correlation was observed in PDL1 expression at the optimum cut-off value between male and female cases (*p* = 0.04) as shown in Table 1. However, the PDL1 level association with other clinico-pathological variables was equivocal.

#### 3.2.2. CA19-9

In our study, there were 6 (31.5%) cases with liver metastasis and 13 (68.4%) were without metastasis. Unlike PDL1, the expression of CA19-9 significantly correlated among cases with, and without, evidence of metastasis (*p* = 0.0043). However, CA19-9 showed no significant correlation between males and females as seen in PDL1. The CA19-9 was directly proportional to the tumor size and showed a significant (*p* = 0.004) clinical association with the size of the tumor as shown in Table 1. No other statistically significant association was found between clinical characteristics and the CA19-9 concentration level.

### 3.3. Correlation of PDL1 and CA19-9 Concentrationsin Cancer Cases and Inflammatory Lesion

**Figure 2:**
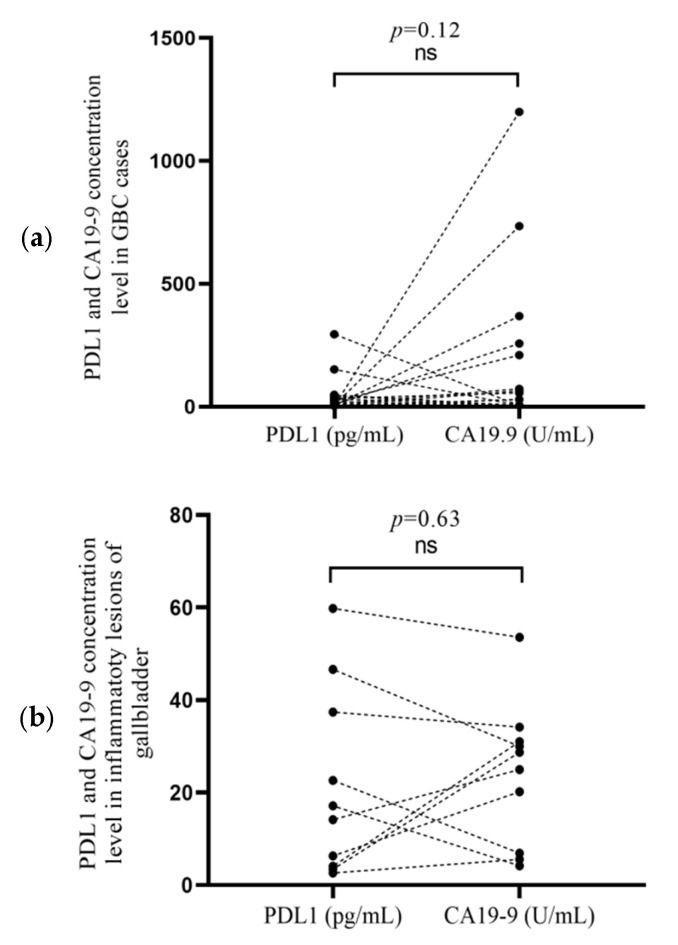
(**a**) PDL1 and CA19-9 concentration level in GBC cases (*n* = 19), (*p* = 0.12); (**b**) PDL1 and CA19-9 concentration level in inflammatory lesions of gallbladder (*n* = 10), (*p* = 0.63).

The concentrations of PDL1 and CA19-9 were compared among GBC cases (*n* = 19) and inflammatory lesions (*n* = 10) as shown in Figure 2. The PDL1 concentration was relatively low compared to CA19-9 among cancer cases (*p* = 0.12) as shown in Figure 2a. However, when the PDL1 concentration was compared to CA19-9 among inflammatory lesions, the levels of concentration showed approximate proportional values without statistical significance (*p* = 0.63) as shown in Figure 2b.

### 3.4. Correlation of PDL1 Protein Concentration with Relative PDL1 mRNA Expression

**Figure 3:**
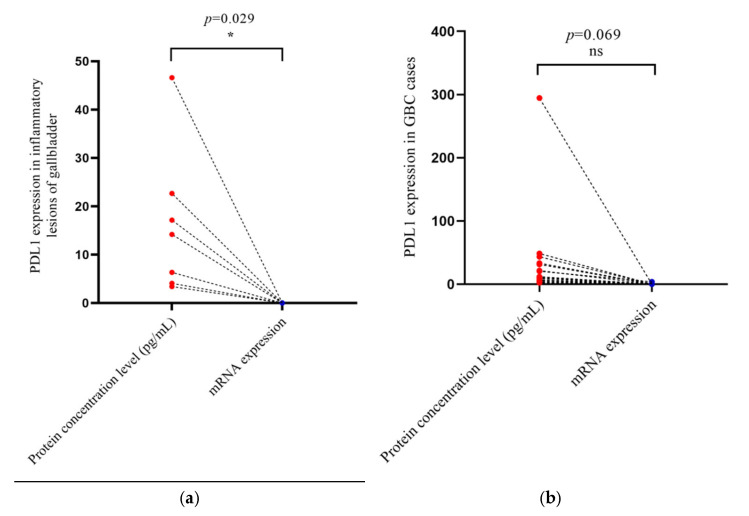
(**a**) PDL1 protein concentration level and its relative mRNA expression in inflammatory lesions (*n* = 7), (*p* = 0.029); (**b**) PDL1 protein concentration level and its relative mRNA expression in cancer cases (*n* = 17), (*p* = 0.069). The red color dot represents protein concentration in individual cancer cases and the blue color dot represents mRNA expression in individual inflammatory cases of gallbladder.

The PDL1 protein concentration was compared with its relative mRNA expression among the matched 24 cases which included 17 cancer and 7 inflammatory cases (Figure 3). It was observed that the fold change concentration level of the PDL1 protein was relatively high in comparison to the corresponding fold change level expression of mRNA. The lesser fold change level in RNA expression yielded more protein fold changes. A significant correlation was found between the PDL1 protein and its relative RNA expression in 7 inflammatory cases (*p* = 0.029) as shown in Figure 3a, whereas no significant correlation was found in cases of GBC (*p* = 0.069), as shown in Figure 3b.

### 3.5. Association of PDL1 and CA19-9 Concentration with Overall Survival

**Figure 4:**
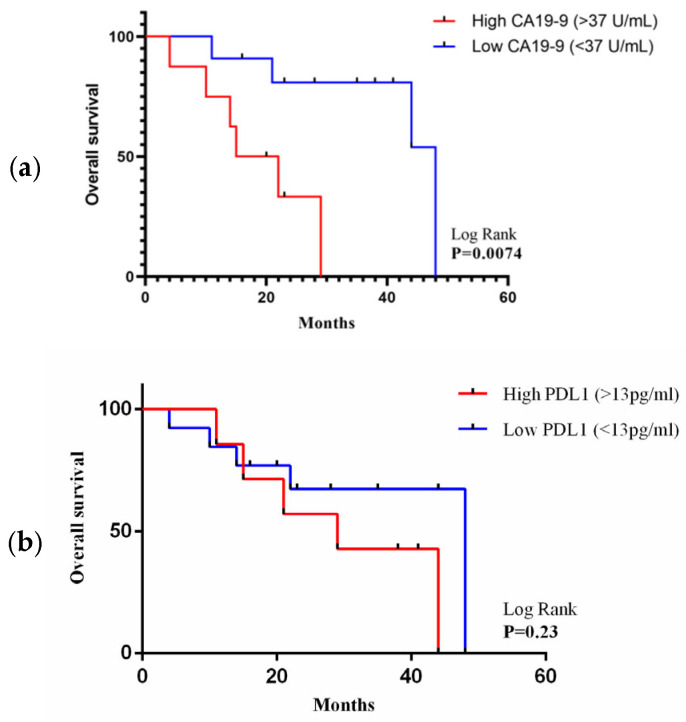
(**a**) Kaplan–Meier survival curve of CA19-9 expression (cut-off value of 37 U/mL, *p* = 0.007). (**b**) Kaplan–Meier survival curve of PDL1 expression (cut-off value of 13 pg/mL, *p* = 0.23).

A Kaplan–Meier survival curve analysis was completed in GBC cases (*n* = 19) in comparison with PDL1 and CA 19-9 concentrations using log-rank testing. Overall survival (OS) was defined as the time from the patient undergoing surgery to death. Follow-up was ended on 30 October 2022, and the study’s median follow-up for overall survival was 23 months. An optimum concentration cut-off value (37 U/mL) of CA19-9 was used for predicting overall survival with high/low CA19-9 expression. A significant (*p* = 0.0074) poor overall survival was observed in cases having high CA19-9 expression when compared to cases having low CA19-9 expression, as shown in Figure 4a. Similarly, an optimum concentration cut-off value (13.03 pg/mL) of PDL1 was used for the survival analysis. It was observed that a high PDL1 concentration in cancer cases had poor overall survival compared to cases with a low PDL1 concentration, but with no statistical significance (*p* = 0.23), as shown in Figure 4b.

### 3.6. Univariate and Multivariate Analysis of Prognostic Factors

The univariate and multivariate analyses of prognostic factors to predict overall survival are shown in Table 2. According to the univariate analysis, the CA19-9 concentration level (<37 U/mL vs. >37 U/mL), liver metastasis (with vs. without metastasis), and tumor size (T1/T2 vs. T3/T4) showed a significant correlation with poor overall survival (*p* = 0.0424, *p* = 0.0102, and *p* = 0.0041, respectively). All other prognostic factors were found to be nonsignificant in the overall survival prognosis. However, the abovementioned variables were not significantly correlated in the multivariate analysis to predict prognosis in GBC patients.

## 4. Discussion

The previously published two studies [17,20] aimed to check the prognostic effect of PDL1 expression in gallbladder cancer tissue through the immunohistochemistry (IHC) technique. Both the studies concluded in a contradicting manner to each other in terms of the prognostic value of PDL1 expression on overall survival in cancer cases. The first study by Neyaz et al. [20] claimed that PDL1 was not a promising prognostic marker and had no influence on the overall survival of GBC cases. However, Kim et al. [17] indicated that the overexpression of PDL1 in GBC was associated with a negative prognostic impact. Until now, no published study has aimed to evaluate comparative PDL1 concentration levels in cancer and inflammatory lesions with a primary objective of targeted therapy in GBC. To the best of our knowledge, there are no data on the protein lysate-based expression of PDL1 and its corresponding mRNA expression in both cancerous as well as inflammatory lesions of the gallbladder. This is probably one of the unique kinds of studies where the PDL1 protein concentration in two phenotypes of gallbladder tissue (cancer and inflammatory lesion) is being studied. A comparable high concentration of the PDL1 level in cancer was observed when compared to inflammatory lesions, but as such no significant (*p* = 0.48) association was found, which may be due to the smaller recruitment of inflammatory lesions, or due to the limited number of participants. The cut-off value of PDL1 concentration is not well established due to the paucity of published data both on clinical practice and trials on GBC. Hence, based on the ROC curve, the optimum PDL1 concentration cut-off value of 13.03 pg/mL was determined in our study. The cut-off value was then correlated with clinico-pathological features.

The study published by Sachan et al. [21] evaluated the prognostic effect of CA19-9 and CEA in gallbladder cancer and found that CA19–9 was better than CEA in the prediction of tumor burden and in predicting recurrence. Furthermore, the Bind et al. [22] study assessed CA19-9 and CA125 concentration levels for diagnosis and evaluation in gallbladder cancer. They also concluded CA19-9 was a much better prognostic marker than the CA125 level [22]. There is no study published to date which has mutually evaluated PDL1 and CA19-9 protein concentrations in cancer cases and inflammatory lesions of the gallbladder. As withPDL1, a comparable high concentration was also observed in CA19-9 in cancer cases when compared to inflammatory lesions with no significance (*p* = 0.17) (as shown in Figure 1b). A validated optimum CA19-9 concentration cut-off value of 37 U/mL was used for the clinico-pathological correlation in conjunction with the CA19-9 concentration.

In the clinico-pathological correlation of GBC/inflammatory cases with the PDL1 concentration level, there were 14/29 (48.2%) cases with a PDL1 concentration level above the optimum cut-off value, whereas the remaining 15 cases (51.7%) had a PDL1 concentration level below the optimum cut-off value. In the current study, there were 11 (57.8%) cases presented with T1/T2, while 8(42.1%) were at T3/T4 with significance (*p* = 0.96). There was no significant association of PDL1 with tumor size; however, it showed a significant association withCA19-9. However, one of the Kim et al. [17] studies had shown the IHC-based PDL1 correlation with tumor size and found it to be statistically significant when compared with tumor size. These findings may not be exactly revalidated in our experiments, as our study is based on protein lysate-based PDL1 expression. The Kim et al. [17] study also carried an observation of biased error in the interpretation of IHC. Another IHC-based study by Albrecht et al. [23] also correlated PDL1 expression with clinical stages of cases (*p* = 0.22), which was also not statistically significant.

The Neyaz et al. [20] study revealed that PDL1 expression has a positive significant (*p* = 0.051) correlation with lymph node metastasis in gallbladder cancer cases. The Kim et al. [17] study showed a correlation between PDL1 expression in tumor cells at cut-off levels of 1% (*p* = 0.260), 10% (*p* = 0.137), and 50% (*p* = 0.093) with lymph node metastasis. We also correlated lymph node metastasis with the PDL1 concentration level in our study and found that there were 6 (31.5%) cases presented with lymph node metastasis, and 13 (68.4%) were without any metastasis and had no significant (*p* = 0.59) association. The authors Kim et al. [17] evidenced that histopathological grades (well-differentiated/moderately differentiated/poorly differentiated) had a significant positive correlation in gallbladder tumor cells with PDL1 expression at cut-off values *of* 1% (*p* = 0.001), 10% (*p* = 0.001), and 50% (*p* = 0.001). In our study, there were 17/19 cases (89.4%) which were morphologically characterized as well-differentiated adenocarcinoma, but 2 (10.5%) cases were poorly differentiated/undifferentiated carcinoma and had no significance (*p* = 0.079). 

As per the published literature, it appears that females are quite predisposed to the development of gallstone disease (GSD) and eventually converting to GBC. The probable explanation to this could be that female sex hormones play an important role in the functioning of the gallbladder [23,24,25]. The risk of GSD development is increased by an early age pregnancy, menarche at a young age, and prolonged fertility as a consequence of prolonged hormonal exposure. It is assumed that estrogen and progesterone are involved in the pathogenesis of GBC and GSD [26,27,28]. Gupta et al. [13] analyzed the estrogen receptor (ER) and progesterone receptor (PR) expression in both benign and malignant tissues of the gallbladder through IHC. The study revealed that ER expression was significantly higher in GBC compared to the inflammatory lesion (benign), whereas PR expression showed no significant difference in both conditions (benign, 40%; malignant, 52%). This study also suggested that ER and PR may act as potential anti-hormonal therapy for gallbladder cancer. Bharathi et al. [29] also aimed to check the ER and PR expression in 47 cases of GBC through the IHC method, and observed that both were simultaneously expressed in a significant proportion (23.4%) of GBC patients. This study further found out that receptor expression correlated with metaplasia, dysplasia, and the early stage of a tumor, whereas nonexpression of the receptor correlated with the metastatic stage. Srivastava et al. [30] explored the role of genetic variants in estrogen (*ER1, ER2*) and progesterone (*PR*) receptors in GBC through the PCR-RFLP method, and showed the role of ER1IVS1-397C>T, ER1IVS1-351A>G, and ER2-789 A>C variants in GBC.

There are few studies available on breast cancer and non-small cell lung cancer (NSCLC) stating that PDL1 expression is responsive to hormonal profiles. Shuai et al. used the breast cancer dataset through the TCGA database (The Cancer Genome Atlas) and compared thePD1/PDL1 levels in breast cancer cases. This study evaluated the expression of the IL-17-induced increased expression of PD1/PDL1 among ER-negative and triple negative cases of breast cancer (TNBC), and identified that PD1/PDL1 increased with the decreased expression of ER. They concluded that PD1/PDL1 expression decreased with the increase in estrogen receptor levels in breast cancer among both ER-negative and TNBC cases [31]. In another study, Manson et al. checked the prognostic values ofPD1/PDL1 expression in 164 male breast cancer and 247 female breast cancer cases through IHC. They identified that PD1/PDL1 expression was correlated with only grade 3 tumors in male breast cancer. However, female breast cancer cases showed a significant correlation in PDL1 expression with ER and PR negativity and with more aggressive molecular subtypes of cancer (Luminal, Her 2driven, and Triple negative). This study concluded that PDL1 immunotherapy would be more responsive to female breast cancer patients in comparison to male breast cancer patients due to the more hormonal imbalance in female estrogen and progesterone profiles [32]. In another study, Skov et al. used 108 NSCLC patients(46 female and 62 male)toevaluate the effect of sex hormones on PDL1 expression through ELISA. The results showed that the serum sPD1 level was significantly (*p* = 0.048) higher in female cases when compared to male cases. The authors of this study also evaluated the mPD1 expression level and found amore significant (*p* = 0.0431) level of expression in female cases than that of male cases [33].

In our study, PDL1 showed a significant correlation between females and males (*p* = 0.04). However, Albrecht et al. [34] found no correlation in PDL1 expression with the gender of gallbladder cancer cases [34]. Yet, one of the published meta-analyses on gallbladder cancer claimed that the female hormone has a significant impact on the disease incidence rate [13]. Though our study has a limited number of participants, the female predilection of GBC is still evident in our data.

We have also compared cases with local liver metastasis (31.5%) and without liver metastasis (68.4%) but found no significant association. After a thorough dissection of all the published manuscripts, we could not find any published data on the association between PDL1 and liver metastasis. The possible reason for this lack of data is that many researchers consider liver metastasis as a local invasion, especially in the case of GBC, owing to its proximity to the liver.

The prognostic impact of CA19-9 is already well-established, and we also found similar results in our cohort. In our study, we compared CA19-9 expression in both cancer cases and inflammatory lesions of the gallbladder. There were 6 (31.5%) cases with liver metastasis, and 13(68.4%) were without metastasis. Unlike PDL1, the expression of CA19-9 was significantly correlated among cases with and without evidence of metastasis (*p* = 0.0043). However, CA19-9 showed no significant correlation between males and females, like in PDL1. The CA19-9 was directly proportional to the tumor size and showed a significant (*p* = 0.004) clinical association. These results concluded that an increase in the CA19-9 level indicates high metastatic potential and increased tumor burden. No other statistically significant association was found between clinical characteristics and CA19-9 expression.

We conducted a mutually exclusive analysis of thePDL1 and CA19-9 protein concentrations in 19 cancer cases and 10 inflammatory lesions of the gallbladder (*p* = 0.48 and *p* = 0.17), respectively, as shown in Figure 1. We observed a relatively low PDL1 concentration among GBC (*p* = 0.12) compared to the CA19-9 level, as shown in Figure 2a. However, inflammatory cases showed approximate proportional values of the PDL1 concentration and CA19-9 concentration level without statistical significance (*p* = 0.63), as shown in Figure 2b. PDL1, being an immunomodulator receptor, may give an additional alternative treatment option to cases; however, its receptors appear to be more prevalent in inflammatory lesions due to the possible cross-talk between immunity and inflammation. The concentration of PDL1 was not high in cancerous cases as immortalized malignant cells might be escaping the immune checkpoints to proliferate as a general universal phenomenon. There are equivocal studies based on IHC on the PDL1 expression on gallbladder carcinoma with one supporting expression [17] while the other is not [20]. The comparison of the current study may be difficult with the previously published studies, as it is based on an ELISA-based estimation of PDL1 in the protein lysate of GBC.

In our study, we also tried to find the correlation of PDL1 mRNA expression with its protein concentration, as shown in Figure 3. The key assumption in evaluating the gene mRNA expression is that it may predict its protein expression. It was observed that the fold change concentration level of thePDL1 protein was relatively high in comparison to the corresponding fold change level expression of mRNA. The lesser fold change level change in RNA expression yielded more protein fold changes.

These results showed that a protein-based assay for PDL1 expression would be more sensitive for GBC risk stratifications because of the higher resolution in the form of higher fold changes, while this differentiation may be missed in mRNA-based assays owing to the low resolution in the form of low fold changes. 

Our results showed a significant positive correlation was found between the PDL1 protein and its relative mRNA expression in 7 inflammatory cases (*p* = 0.029), as shown in Figure 3a, whereas no significant correlation was found in the cases of GBC (*p* = 0.069), as shown in Figure 3b. There is one published meta-analysis that discusses RNA vs. protein interaction [35]. However, no specific studies have explored the RNA–protein correlation on PDL1 in human tissues. This is the first time that PDL1 protein concentration was correlated with its corresponding mRNA expression. 

In our study, a Kaplan–Meier survival analysis identified a comparable poor overall survival in cancer cases having a high PDL1 concentration in comparison with low PDL1 concentration, but with no statistically significant association (*p* = 0.23) as shown in Figure 4b. These initial results are encouraging and may still need more samples to be validated further. Nevertheless, this finding promotes a therapeutic strategy for personalized immunotherapy, especially GBC cases which are not eligible for surgical interventions. Few of the previous studies showed a poor prognosis with high PDL1 concentration levels [36,37]. Similarly, a significant (*p* = 0.0074) poor overall survival association was found in GBC cases having a high CA19-9 expression when compared to cases having a low CA19-9 expression as shown in Figure 4a. These results follow the previously published studies [38,39].

The current studyas per the univariate and multivariate Cox proportional hazards regression analyses, CA19-9 concentration level (<37 U/mL vs. >37 U/mL), liver metastasis (with vs. without metastasis), and tumor size (T1/2 vs. T3/4) showed a significant positive correlation with poor overall survival (*p* = 0.0424, *p* = 0.0102, *p* = 0.0041, respectively). Kim et al. [17] also claimed that CA19-9 is an independent prognostic marker for overall survival in gallbladder cancer patients [17]. Similar findings were observed by Higuchi et al. [40], wherea univariate and multivariate analysis of tumor size (T1/2 vs. T3/4) with overall survival revealed a significant worse prognosis in cancer cases (*p* = 0.015, *p* = 0.007) [40].

There is one ongoing phase 2 clinical trial that aims to investigate the efficacy, safety, pharmacokinetics, and pharmacodynamics of STI-3031 (anti-PDL1 antibody) in patients with selected relapsed or refractory (R/R) malignancies of the biliary tract(intrahepatic cholangiocarcinoma, extrahepatic cholangiocarcinoma, or gallbladder cancer), extra nodal NK/T-cell lymphoma (ENKTL), peripheral T-cell lymphomas (PTCL), and diffuse large B cell lymphoma [41].

This study investigated the PDL1 and CA19-9 marker expressions in both cancer and inflammatory cases of the gallbladder. The PDL1 protein and its corresponding mRNA correlation study were performed to highlight the higher sensitivity of protein-based assays for PDL1 expression relative to RNA-based assays. By virtue of the fact that the fold change concentration levels of the PDL1 protein were relatively higher compared to the fold change level expression of mRNA. The minor fold change expression is resolved better in protein based assays which might remain undetected in RNA based assays. The univariate and multivariate analyses of prognostic factors were to predict the overall survival of the cases with the high expression of CA19-9 and PDL1.

There were a few limitations of the present study. First, the sample size used in the study was relatively small. The GBC cases eligible for surgical resections were very few owing to delayed presentations. Second, only biological investigations were being used for comparisons. The inclusion of imaging findings would have improved the confidence of inference. A future study with a larger number of paired samples with same (rather than independent inflammatory control) malignant versus inflammatory parts of the resected gallbladder, along with long-term follow-up with the inclusion of relapsing cases may provide a deeper insight.

## 5. Conclusions

This study, though conducted on a limited number of cases, still highlights the potential of PDL1 and CA19-9 as a probable predictor of a poor prognosis in GBC cases. This is an initial observation only and may need further validation in a larger cohort. The PDL1 inhibitors may act as potential therapeutic targets for GBC, especially for those who are not eligible for any surgical intervention. This may ultimately improve the poor overall survival of GBC cases. Our results showed that a protein-based assay for PDL1 expression would be more sensitive compared to RNA based assays for GBC risk stratifications.

## Figures and Tables

**Table 1:** Patients’ clinico-pathological characteristics correlated with PDL1 and CA19-9 concentration at their respective optimum cut-off values (13 pg/mL and 37 U/mL), (*n* = 29).

Characteristics		PDL1		*p* Value	CA19-9		*p* Value
		Low Expression (≤13.03 pg/mL)	High Expression (≥13.03 pg/mL)		Low Expression (≤37 U/mL)	High Expression (≥37 U/mL)	
Age above 60		2 (33.3%)	4 (66.6%)	0.31	3 (50%)	3 (50%)	0.25
Age below 60		13 (56.5%)	10 (43.4%)		17 (73.91%)	6 (26.09%)	
Liver metastasis		4 (66.6%)	2 (33.3%)	0.59	1 (16.67%)	5 (83.33%)	0.0043 *
Liver without metastasis		7 (53.8%)	6 (46%)		11 (84.62%)	2 (15.38%)	
Gender	Female	11 (68.75%)	5 (31.25%)	0.04 *	11 (68.75%)	5 (31.25%)	0.97
	Male	4 (31%)	9 (69%)		9 (69.23%)	4 (30.77%)	
Lymph node metastasis	N0	8 (61.5%)	5 (38.4)	0.82	9 (64.29%)	5 (35.71%)	0.3
	N1	4 (66.6%)	2 (33.3%)		2 (40%)	3 (60%)	
Differentiation	Well/moderate	11 (64.7%)	6 (35.2%)	0.0796	9 (52.9%)	8 (47.05%)	0.2
	Poor	0 (0%)	2 (100%)		2 (100%)	0	
Tumor size	T1	2 (100%)	0 (0%)	0.96	2 (100%)	0	0.004 *
	T2	4 (44.4%)	5 (55.5%)		8 (88.8%)	1 (11.1%)	
	T3	4 (66.6%)	2 (33.3%)		0	6 (100%)	
	T4	1 (50%)	1 (50%)		1 (50%)	1 (50%)	

*p* < 0.05 * is statistically significant.

**Table 2:** Univariate and multivariate analyses of prognostic factors.

Characteristics		*n*	Univariate Cox Regression		Multivariate Cox Regression	
			Hazard Ratio [95CI]	*p* Value	Hazard Ratio	*p* Value
PDL1 concentration level	<13 pg/mL	11	0.6672 [0.1669–2.384]	0.615	0.913 [0.1194–6.440]	0.9250
	>13 pg/mL	8				
CA19-9 concentration level	<37 U/mL	11	3.875 [1.065–15.76]	0.0424 *	1.073 [0.05903–13.64]	0.9588
	>37 U/mL	8				
Lymphnode metastasis	without metastasis	13	3.081 [0.8462–11.22]	0.0785	3.416 [0.8119–16.13]	0.0938
	with metastasis	6				
Differentiation	poor	2	0.9740 [0.1809–18.03]	0.9801	0.9785 [0.04478–43.82]	0.9894
	well/moderate	17				
Liver metastasis	without metastasis	13	7.159 [1.645–36.96]	0.0102 *	8.185 [0.8095–154.5]	0.1131
	with metastasis	6				
Age	<60	4	1.699 [0.3638–6.167]	0.4452	1.374 [1.078–12.67]	0.7846
	>60	15				
Tumor size	T1/T2	11	7.692 [2.045–36.82]	0.0041 *	-	-
	T3/T4	8				

*p* < 0.05 * is statistically significant.

## Data Availability

The data presented in this study are available on reasonable request from the corresponding and first author.

## References

[1] Mehrotra B. Gallbladder Cancer: Epidemiology, Risk Factors, Clinical Features, and Diagnosis. Up Date 2013. https://medilib.ir/uptodate/show/2491.

[2] Goetze T.O. (2015). Gallbladder carcinoma: Prognostic factors and therapeutic options. World J. Gastroenterol..

[3] Lai C.H.E., Lau W.Y. (2008). Gallbladder cancer—A comprehensive review. Surgeon.

[4] Goldin R.D., Roa J.C. (2009). Gallbladder cancer: A morphological and molecular update. Histopathology.

[5] Albores-Saavedra J., Tuck M., McLaren B.K., Carrick K.S., Henson D.E. (2005). Papillary carcinomas of the gallbladder: Analysis of noninvasive and invasive types. Arch. Pathol. Lab. Med..

[6] Hundal R., Shaffer E.A. (2014). Gallbladder cancer: Epidemiology and outcome. Clin. Epidemiol..

[7] Dutta U., Bush N., Kalsi D., Popli P., Kapoor V.K. (2019). Epidemiology of gallbladder cancer in India. Chin. Clin. Oncol..

[8] Roa I., Ibacache G., Roa J., Araya J., De Aretxabala X., Muñoz S. (2006). Gallstones and gallbladder cancer-volume and weight of gallstones are associated with gallbladder cancer: A case-control study. J. Surg. Oncol..

[9] Wistuba I.I., Gazdar A.F. (2004). Gallbladder cancer: Lessons from a rare tumour. Nat. Rev. Cancer.

[10] Pilgrim C.H.C., Groeschl R.T., Christians K.K., Gamblin T.C. (2013). Modern perspectives on factors predisposing to the development of gallbladder cancer. HPB.

[11] Hsing A.W., Bai Y., Andreotti G., Rashid A., Deng J., Chen J., Goldstein A.M., Han T.-Q., Shen M.-C., Fraumeni J.F. (2007). Family history of gallstones and the risk of biliary tract cancer and gallstones: A population-based study in Shanghai, China. Int. J. Cancer.

[12] Konstantinidis I.T., Deshpande V., Genevay M., Berger D., Castillo C.F.-D., Tanabe K.K., Zheng H., Lauwers G.Y., Ferrone C.R. (2009). Trends in presentation and survival for gallbladder cancer during a period of more than 4 decades: A single-institution experience. Arch. Surg..

[13] Gupta P., Agarwal A., Gupta V., Singh P.K., Pantola C., Amit S. (2012). Expression and clinicopathological significance of estrogen and progesterone receptors in gallbladder cancer. Gastrointest. Cancer Res..

[14] Ghosh M., Sakhuja P., Singh S., Agarwal A. (2013). P53 and beta-catenin expression in gallbladder tissues and correlation with tumor progression in gallbladder cancer. Saudi J. Gastroenterol..

[15] Okada K.-I., Kijima H., Imaizumi T., Hirabayashi K., Matsuyama M., Yazawa N., Dowaki S., Tobita K., Ohtani Y., Tanaka M. (2012). Clinical significance of wall invasion pattern of subserosa-invasive gallbladder carcinoma. Oncol. Rep..

[16] Song X., Hu Y., Li Y., Shao R., Liu F., Liu Y. (2020). Overview of current targeted therapy in gallbladder cancer. Signal Transduct. Target. Ther..

[17] Kim J.H., Kim K., Kim M., Kim Y.M., Suh J.H., Cha H.J., Choi H.J. (2020). Programmed death-ligand 1 expression and its correlation with clinicopathological parameters in gallbladder cancer. J. Pathol. Transl. Med..

[18] Fluss R., Faraggi D., Reiser B. (2005). Estimation of the Youden Index and its associated cutoff point. Biom. J..

[19] Xing H., Wang J., Wang Y., Tong M., Hu H., Huang C., Li D. (2018). Diagnostic value of CA 19-9 and carcinoembryonic antigen for pancreatic cancer: A meta-analysis. Gastroenterol. Res. Pract..

[20] Neyaz A., Husain N., Kumari S., Gupta S., Shukla S., Arshad S., Anand N., Chaturvedi A. (2018). Clinical relevance of PDL1 expression in gallbladder cancer: A potential target for therapy. Histopathology.

[21] Sachan A., Saluja S.S., Nekarakanti P.K., Nimisha, Mahajan B., Nag H.H., Mishra P.K. (2020). Raised CA19-9 and CEA have prognostic relevance in gallbladder carcinoma. BMC Cancer.

[22] Bind M., Mishra R., Kumar V., Misra V., Singh P. (2021). Serum CA 19-9 and CA 125 as a diagnostic marker in carcinoma of gallbladder. Indian J. Pathol. Microbiol..

[23] Chen A., Huminer D. (1991). The role of estrogen receptors in the development of gallstones and gallbladder cancer. Med. Hypotheses.

[24] Singletary B.K., van Thiel D.H., Eagon P.K. (1986). Estrogen and progesterone receptors in human gallbladder. Hepatology.

[25] Hould F.S., Fried G.M., Fazekas A.G., Tremblay S., Mersereau W.A. (1988). Progesterone receptors regulate gallbladder motility. J. Surg. Res..

[26] Dalgnault P.G., Fazekas A.G., Rosenthall L., Fried G.M. (1988). Relationship between gallbladder contraction and progesterone receptors in patients will gallstones. Am. J. Surg..

[27] Baskaran V., Vij U., Sahni P., Tandon R.K., Nundy S. (2005). Do the progesterone receptors have a role to play in gallbladder cancer?. Int. J. Gastrointest. Cancer.

[28] Nakamura S., Muro H., Suzuki S. (1989). Estrogen and progesterone receptors in gallbladder cancer. Jpn. J. Surg..

[29] Saranga Bharathi R., Singh R., Gupta R., Verma G.R., Kalra N., Kiran K., Joshi K. (2015). Female Sex Hormone Receptors in Gallbladder Cancer. J. Gastrointest. Cancer.

[30] Srivastava A., Sharma K.L., Srivastava N., Misra S., Mittal B. (2012). Significant role of estrogen and progesterone receptor sequence variants in gallbladder cancer predisposition: A multi-analytical strategy. PLoS ONE.

[31] Shuai C., Yang X., Pan H., Han W. (2020). Estrogen Receptor Downregulates Expression of PD1/PDL1 and Infiltration of CD8+ T Cells by Inhibiting IL-17 Signaling Transduction in Breast Cancer. Front. Oncol..

[32] Manson Q.F., ter Hoeve N.D., Buerger H., Moelans C.B., van Diest P.J. (2018). PD-1 and PDL1 Expression in Male Breast Cancer in Comparison with Female Breast Cancer. Target. Oncol..

[33] Gu Y., Tang Y.Y., Wan J.X., Zou J.Y., Lu C.G., Zhu H.S., Sheng S.Y., Wang Y.F., Liu H.C., Yang J. (2022). Sex difference in the expression of PD-1 of non-small cell lung cancer. Front. Immunol..

[34] Albrecht T., Brinkmann F., Albrecht M., Lonsdorf A., Mehrabi A., Hoffmann K., Kulu Y., Charbel A., Vogel M., Rupp C. (2021). Programmed death ligand-1 (Pdl1) is an independent negative prognosticator in western-world gallbladder cancer. Cancers.

[35] Guo Y., Xiao P., Lei S., Deng F., Xiao G.G., Liu Y., Chen X., Li L., Wu S., Chen Y. (2008). How is mRNA expression predictive for protein expression? A correlation study on human circulating monocytes. Acta Biochim. Biophys..

[36] Lin J., Long J., Wan X., Chen J., Bai Y., Wang A., Yang X., Wu Y., Robson S.C., Sang X. (2018). Classification of gallbladder cancer by assessment of CD8+ TIL and PDL1 expression. BMC Cancer.

[37] Lei C., Peng X., Gong X., Fan Y., Wu S., Liu N., Li L., Huang J., Zheng G., Long Z. (2019). Prognostic role of programmed death-ligand 1 expression in patients with biliary tract cancer: A meta-analysis. Aging.

[38] Wen Z., Si A., Yang J., Yang P., Yang X., Liu H., Yan X., Li W., Zhang B. (2017). Elevation of CA19-9 and CEA is associated with a poor prognosis in patients with resectable gallbladder carcinoma. HPB.

[39] Ballehaninna U.K., Chamberlain R.S. (2012). The clinical utility of serum CA 19-9 in the diagnosis, prognosis and management of pancreatic adenocarcinoma: An evidence based appraisal. J. Gastrointest. Oncol..

[40] Higuchi R., Yazawa T., Uemura S., Matsunaga Y., Ota T., Araida T., Furukawa T., Yamamoto M. (2020). Examination of prognostic factors affecting long-term survival of patients with stage 3/4 gallbladder cancer without distant metastasis. Cancers.

[41] ClinicalTrials. https://ClinicalTrials.gov.

